# Radiotherapy dose prediction using off‐the‐shelf segmentation networks: A feasibility study with GammaPod planning

**DOI:** 10.1002/mp.17711

**Published:** 2025-02-28

**Authors:** Qingying Wang, Mingli Chen, Mahdieh Kazemimoghadam, Zi Yang, Kangning Zhang, Xuejun Gu, Weiguo Lu

**Affiliations:** ^1^ Department of Radiation Oncology The University of Texas Southwestern Medical Center Dallas Texas USA; ^2^ Department of Radiation Oncology Stanford University Stanford California USA

**Keywords:** deep learning, dose prediction, radiation therapy

## Abstract

**Background:**

Radiotherapy requires precise, patient‐specific treatment planning to achieve high‐quality dose distributions that improve patient outcomes. Traditional manual planning is time‐consuming and clinically impractical for performing necessary plan trade‐off comparisons, including treatment modality selection, prescription dose settings, and organ at risk (OAR) constraints. A time‐efficient dose prediction tool could accelerate the planning process by guiding clinical plan optimization and adjustments. While the deep convolutional neural networks (CNNs) are prominent in radiotherapy dose prediction tasks, most studies have attempted to customize network architectures for different diseases and treatment modalities.

**Purpose:**

This study proposes a universal and efficient strategy, Seg2Dose, leveraging a state‐of‐the‐art segmentation network for radiotherapy dose prediction without the need for model architecture modifications. We aim to provide a convenient off‐the‐shelf dose prediction tool that simplifies the dose prediction process, enhancing planning speed, and plan quality while minimizing the need for extensive coding and customization.

**Methods:**

The proposed Seg2Dose consists of three modules: the Adapter, the segmentation network, and the Smoother. Prior to model training, the Adapter processes dose distributions into dose level map with an adjustable interval, which serves as the ground truth of the segmentation network, and generates two input channels: weighted avoidance image and normalized prescribed dose image. The segmentation network predicts dose levels from input channels using the nnU‐Net, which was trained, validated and tested on 304, 77, and 64 breast cancer GammaPod treatment plans from 90 patients. The Smoother converts the predicted dose levels into continuous dose distribution with a Gaussian filter. The performance of Seg2Dose models with two different dose level intervals, 2% (Seg2Dose 2%) and 5% (Seg2Dose 5%), was evaluated by the Dice similarity coefficients (DSCs), voxel‐based mean absolute percent error (MAPE), dose‐volume histogram (DVH) metrics, global 3%/2 mm and 3%/1 mm gamma passing rate (GPR), and a case study including normal and worst cases. Additionally, Seg2Dose was compared with an exciting cutting‐edge Cascade 3D (C3D) dose prediction model, which was trained on continuous dose distributions, to investigate the impact of using dose level map.

**Results:**

For dose level prediction, Seg2Dose achieved average DSCs of 0.94 and 0.93 for the 2% and 5% intervals, respectively. For dose distribution prediction, both Seg2Dose 2% and Seg2Dose 5% achieved MAPEs within 6% for targets and most OARs, with the exception of the skin, which had the highest MAPE at 8.58% for Seg2Dose 2% and 15.25% for Seg2Dose 5%. The DVH metrics showed consistent findings. The C3D model has a better performance in GPR than Seg2Dose models. However, the C3D model exhibited higher MAPEs in target areas with lower dose predictions. In the case study, Seg2Dose 2% and C3D predictions were more consistent with clinical plans, showing smaller dose differences compared to Seg2Dose 5%.

**Conclusions:**

Our study confirms the feasibility of leveraging the segmentation network for dose prediction and provides an efficient and off‐the‐shelf approach for dose prediction without requiring extensive coding efforts. This plug‐in tool holds promise for quick dose planning, potentially aiding in the identification of optimal radiotherapy techniques and dosimetric tradeoffs prior to tedious treatment planning.

## 1 INTRODUCTION

Radiotherapy is one of the primary treatments for cancer patients. Before initiating radiation treatment, it is crucial to develop a high‐quality, optimized plan tailored to the specific anatomical structures of patients. With the emergence of diverse treatment modalities and increasingly demanding treatment requirements, modern radiotherapy tends to perform a comprehensive and patient‐specific planning approach, which necessitates planners evaluate multiple factors before designing a plan. These factors include treatment modality selection,^1^, ^2^ prescription dose settings, organ at risk (OAR) constraints, and dosimetric tradeoffs. However, performing plan tradeoff comparison remains a significant challenge in clinical practice because the manual treatment planning is a time‐ and labor‐intensive procedure^3^ that can potentially delay the start of treatment.^4^

Various automatic treatment planning technologies and planning tools have been introduced in recent years.^5^, ^6^, ^7^, ^8^, ^9^ The treatment planning utility based on multi‐criteria optimization (MCO),^10^ which could provide a solution of overviewing all possible plans, navigates the Pareto front of multiple objectives to generate a set of Pareto optimal plans as candidates, allowing the treatment planner to select the most appropriate combination. The obstacle for the MCO‐based planning utility is that the plan generation step is computationally expensive, which requires several hours for complex patient cases to generate a modest number (∼50) of treatment plans.^11^ Although plan generation is parallelizable, the hourly timescale is still unacceptable in the pre‐planning phase. Another automatic treatment planning approach, the dose prediction model based on convolutional neural networks (CNNs),^12^, ^13^, ^14^, ^15^ shows promise as a quick dose preview tool due to its time‐efficient nature. Once the model is well‐trained to learn the potential relationship between anatomical information and optimal dose distribution, it can rapidly predict dose information for a new patient within seconds. Among CNN networks, the segmentation networks with encoder–decoder architecture demonstrate superior performance in predicting patient‐specific voxel‐wise dose distribution, even though their original purpose is to do the segmentation task.^16^ Since their original purpose is to perform semantic segmentation through the final pixel classification layer of the decoder network, several studies have modified the architecture of segmentation networks, such as U‐Net, to customize them for the dose prediction task.^14^, ^15^, ^16^, ^17^

In this study, rather than focusing on the network architecture and learning algorithm, we propose a universal strategy named Seg2Dose to develop an off‐the‐shelf dose prediction tool directly utilizing the segmentation networks. Seg2Dose contains three modules, Adapter, segmentation network, and Smoother. The Adapter is mainly to process and tailor the data into the required discrete format for the segmentation network. The initial prediction of segmentation network is a discrete dose level map. Thus, the Smoother processes the discrete prediction into a continuous dose distribution, making it comparable with clinical plans. The existing dose prediction models typically use computer tomography (CT) images, target and OAR, and beam setup information^18^ as anatomical information for model input.^19^ To improve the generalizability of Seg2Dose, we herein investigate the performance of the model with only two input channels, the normalized prescribed dose image and avoidance region with different weights. The main advantage of the Seg2Dose strategy is that it provides a feasible way to utilize segmentation networks as the black box for the dose prediction task without any coding effort, so researchers can focus more on data preparation, as deep learning is more of a data‐driven approach. Additionally, the performance of Seg2Dose has been compared with the Cascade 3D (C3D) model,^15^ the official winner of the OpenKBP Challenge in 2020, which is trained on continuous dose distribution. With the purpose of enhancing clinical utility, Seg2Dose holds the potential to be further developed into efficient off‐the‐shelf tools, such as a fast dose navigator or treatment modality selector.

## 2 MATERIALS AND METHODS

### 2.1 Patient data

In this retrospective study, we collected a dataset of 90 breast cancer patients, undergoing GammaPod^20^ treatment from 2019 through 2023 at our institution. Ethical approval was granted by the local ethics committee. All treatment plans were manually designed and optimized by the GammaPod treatment planning system (Xcision Medical System, Columbia, MD, USA) with multisource Cobalt‐60 stereotactic radiotherapy. All patients received 5‐fraction treatments with on‐line planning for every fraction. The institutional prescription guideline is 7 Gy, or 8 Gy per fraction to 95% of CTV (clinical target volume) and 6 Gy to > 90% of PTV (planning target volume). For some cases with large (> 100cc) PTV volume, then the prescription is 6 Gy to 95% of PTV. Prior to each fraction, head‐first‐prone CT scans were acquired on either Airo Mobile Intraoperative CT (Brainlab AG, Munich, Germany) or Philips Brilliance Big Bore CT (Philips Healthcare, Amsterdam, Netherlands), both with 120 kVp and scanning lengths covering the whole chest region. CT images had a slice thickness of 1 mm. Pixel resolution varied between 1.17 and 1.37 mm. Targets and 4 OARs, including heart, ribs, skin, and whole breast, were delineated by radiation oncologists/medical physicists.

To prepare the training and validation data for this project, CT images were resampled with 2 mm isotropic resolution and cropped around the mass center of the whole breast to a dimension of 128×128×128. Mask images of targets/OARs and dose images were resampled and cropped in the same way as CT images. The processed plan data were used for model training in this study. We treated each fraction as a separate case and excluded five outlier cases with non‐compliant prescriptions or target hotspots exceeding 102% of the prescription dose. Thus, 90 patients resulted in a total of 445 cases for this study, and we randomly divided the dataset at the patient level into 304 cases (61 patients) for training, 77 cases (16 patients) for validation, and 64 cases (13 patients) for testing, as shown in Table 1.

**TABLE 1:** Three different prescription dose for breast cancer patient treated by GammaPod.

Prescription dose	Training cases	Validation cases	Test cases
6 Gy	12	4	0
7 Gy	194	52	47
8 Gy	98	21	17
Total	304	77	64

### 2.2 Seg2Dose for dose prediction

**FIGURE 1:**
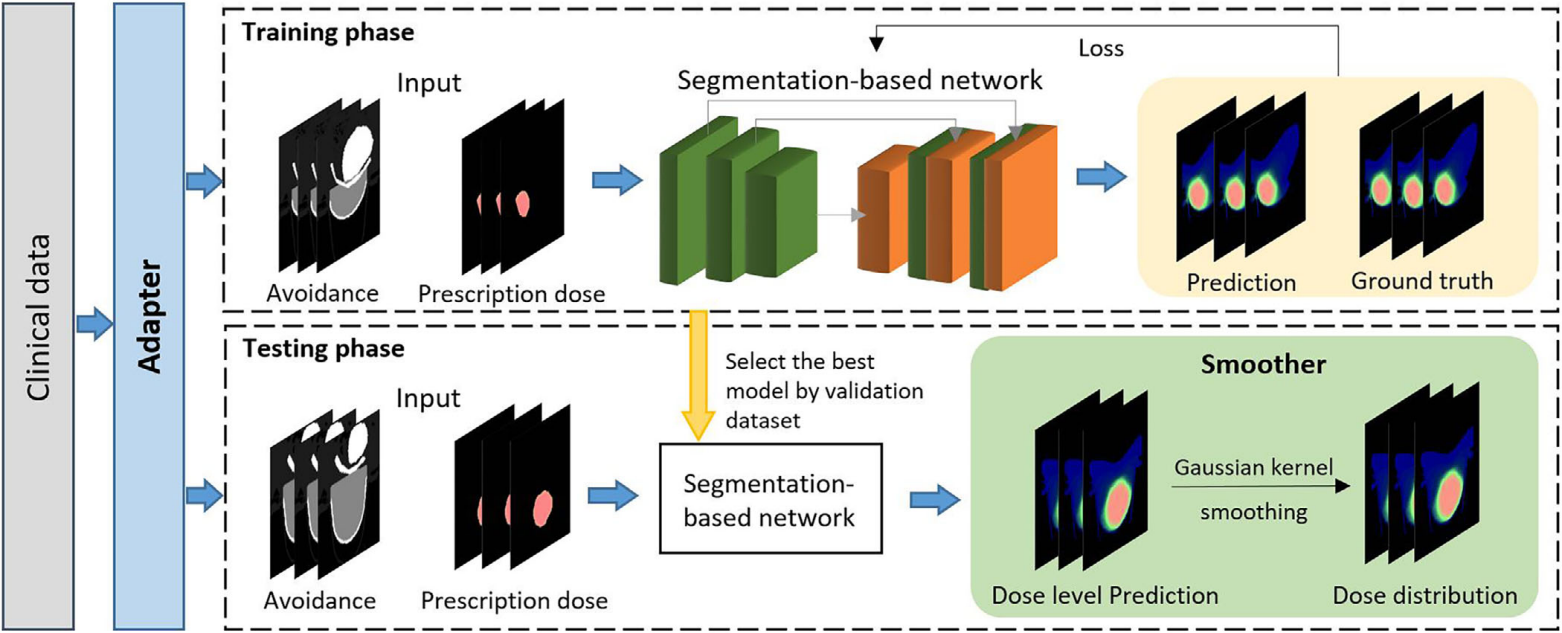
The overview workflow of dose prediction by using the segmentation network.

Figure 1 illustrates the workflow of our proposed Seg2Dose strategy, which consists of three modules: the Adapter, the segmentation network, and the Smoother, for predicting dose distribution. Initially, the Adapter generates two input channels: the avoidance image, which is created from OAR masks with different weights, and the normalized prescribed dose. Additionally, the Adapter discretizes the clinical dose distribution into a dose level map, which serves as the ground truth for model training. Then, an advanced deep learning‐based segmentation network with an encoder–decoder architecture is trained for the dose level prediction task. Here we adopt nnU‐Net^21^ as our segmentation tool, which can be applied out‐of‐the‐box and demonstrates superior performance in the biomedical segmentation domain. To avoid overfitting, we select the model that performs best on the validation set. During the testing phase, the selected model predicts the dose levels based on the input data from the testing set. In the final step, the dose levels are processed by the Smoother, which applies a Gaussian filter to smooth the dose levels into dose distributions, making the predictions comparable to the clinical dose distributions and improving the applicability of the model.

#### 2.2.1 Adapter module

**FIGURE 2:**
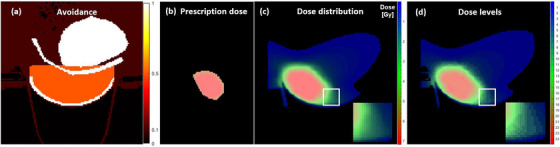
The illustration: (a) the avoidance mask, (b) the normalized prescribed dose, (c) clinical dose distribution (continuous), and (d) discretized dose levels by 5% interval, both with zoomed‐in insets in the same area highlighted by the white square.

The Adapter in the preprocessing phase primarily focuses on generating the input data and aligning the clinical dose distribution with the ground truth format required by the segmentation network. The input data consists of two channels: the avoidance image and the normalized prescribed dose. Traditional methods for training dose prediction models rely on treating OARs as individual inputs, limiting the generalizability and practicality of the model, since clinical datasets are often partially labeled due to patient‐specific considerations.^22^ The avoidance image consolidates multiple OARs and body contours into a single avoidance map and assigns different weights to voxels based on their classification, as illustrated in Figure 2a. The voxel weights were normalized by assigning a value of 1 to the most clinically important weight, representing the highest priority. Specifically, the voxels classified as heart, ribs, and skin are set to 1, those classified as breast are set to 0.5, body voxels are set to 0.1, and the remaining voxels are set to 0. These settings align with the priorities of objectives in the planning optimization process. For the normalized prescribed dose channel, we only consider the dose voxels within PTV as input data. As illustrated in Figure 2b, the prescription dose for the CTV area (the red area) is normalized to 1. For the portion of the PTV that extends beyond CTV (the orange area indicates PTV‐CTV), the prescription dose is normalized relative to the CTV prescription dose. For instance, given the prescription dose for the CTV is 8 Gy and PTV is 6 Gy, the dose in the PTV‐CTV area is normalized to 0.75 Gy. The normalization ensures that the input data is consistent and comparable across different prescription doses. Additionally, the Seg2Dose simplifies the input requirements by considering only the normalized prescribed dose and avoidance regions.

Since segmentation networks are designed to classify and predict discrete data based on predefined categories, we need to discretize the dose distribution into dose levels to effectively train the network for dose prediction tasks. All absolute dose distributions are first normalized to relative dose distributions ranging from 0% to 120%. The granularity of the model predictions depends on the setting of the isodose volume interval. Given the interval i%, the number of isodose volume is T=120%/i%, and the t‐th isodose volume represents the dose range $[\left(t-\frac{1}{2}\right)i\%,\;\left(t+\frac{1}{2}\right)i\%$]. For instance, with a 5% interval, the relative dose distribution from 0% to 120% is divided into 24 isodose volumes, resulting in 24 output channels for the model, as illustrated in Figure 2c,d. Similarly, with a 2% interval, the model has 60 output channels. Smaller intervals lead to more output channels and retain more detailed information.

#### 2.2.2 Smoother module

The initial prediction of the segmentation network is the discrete dose levels; however, a continuous dose distribution is required for clinical practice. After the inference step, Seg2Dose utilizes the Smoother to convert the discretized dose levels into continuous dose distributions by employing a Gaussian filter, which is simple in numerical implementation and widely used in image processing.^23^ The continuous dose distribution D(x) can be generated by applying the Gaussian filter $K_{\sigma }$ to integrate over all y point in the dose level P(y) to obtain the weighted average dose at each point x, as illustrated in Equation (1). The Gaussian filter is defined on the bandwidth σ which determines the degree of smoothing, and Equation (2) shows the expression of n‐dimensional Gaussian kernel. Here we set n=3 for processing 3D dose distribution and σ=2mm to align with the resolution of the dose distribution.

(1) $$D\;\left(x\right)=K_{\sigma }\;*P\;\left(x\right)=\int K_{\sigma }\left(x-y\right)\cdot P\left(y\right)dy\;$$

(2) $$K_{\sigma }\;\left(x\right)=\frac{1}{{\left(2\pi \right)}^{n/2}\sigma^{n}}\;\cdot \exp\left(\frac{-x^{2}}{2\sigma^{2}}\right)$$

### 2.3 Segmentation network and experimental setting

We used the original nnU‐Net architecture without any modifications to its model architecture, parameters, or loss function, as described in the original paper.^21^ The Windows version of nnU‐Net was downloaded from Github (https://github.com/marcus‐wirtz‐snkeos/nnUNet) and compiled with PyInstaller (https://pyinstaller.org/en/stable/) in a development Windows computer with Python environment setup per nnU‐Net requirement. The compiled executables are then copied to a test Windows machine with a single NVIDIA GeForce RTX 2080 Ti GPU and Intel Core i7‐9700K CPU but without Python environment. All nnU‐Net segmentation model training and inference are conducted using the compiled executables on the test computer. And all the original nnU‐Net training parameters are wrapped in the compiled code without any modifications, ensuring it operates as an off‐the‐shelf application. All aspects of the nnU‐Net, including its dynamic adaptation to the dataset, preprocessing, and data augmentation strategies, were maintained as per the original implementation, ensuring the robustness and reproducibility of our results.

To investigate the impact of different intervals i% on the performance of Seg2Dose, the Adapter module was configured with 2% and 5% intervals for model training and testing. The models are referred to as Seg2Dose 2% and Seg2Dose 5%, respectively. The training processes for the Seg2Dose 2% and Seg2Dose 5% models were stopped at 800 epochs with a converged loss score, which took 93 and 28 h, respectively.

The comparison between Seg2Dose and C3D^15^ was conducted to evaluate the effect of discrete dose level training on prediction accuracy. The C3D model treats each OAR as an individual input channel. To adapt the C3D model for the GammaPod dataset, it was modified to include six input channels. The first input channel represents the PTV image, where the voxels within the PTV are assigned values corresponding to their corresponding prescription dose normalized by 6 Gy. The second to fifth input channels are binary masks of four OARs (heart, ribs, skin, and breast). The sixth input channel is the CT image, processed with the clipping and normalization steps from the original C3D model. The output of the C3D is the dose image normalized by 6 Gy. All other components of the C3D model remained unchanged from the original version. The training process for the C3D model was stopped at 24 epochs (12 000 iterations) upon reaching a converged loss score, taking 71 h. The inference times of Seg2Dose and C3D are comparable, with Seg2Dose averaging approximately 1.4 s per case and C3D around 1.88 s per case, measured across the 64 test cases.

### 2.4 Evaluation metrics

To evaluate model performance, the predicted doses for the 64 test cases of 13 patients were compared with doses from the clinical plans. The prediction accuracy of the segmentation model (nnU‐Net) is evaluated by calculating the Dice similarity coefficient (DSC), defined as $\frac{2(A\;\cap \;B)}{A+B}$, where A represents clinical dose levels and B represents predicted dose levels. The DSC is calculated for dose ranges from the first isodose volume to the t‐th isodose volume, corresponding to the range $[\frac{1}{2}i\%,\;\left(t+\frac{1}{2}\right)i\%$]. For the dose distributions generated by Seg2Dose and C3D models, the evaluation process was compared with dose distributions from clinical plans, including statistical comparison of mean absolute percent error (MAPE) in the region of interests (ROIs), various dose‐volume histogram (DVH) metrics with according MAPE and global gamma passing rate (GPR), and quantitative comparison of dose distribution.

Equation (3) provides a general formula for calculating MAPE, applicable to both voxel‐based dose and DVH metrics evaluations. For voxel‐based dose evaluation, MAPE was calculated separately for voxels within a given ROI contour, including the gross target volume (GTV), CTV, PTV, and the four OARs. The calculation of MAPE is based on the smoothed dose distribution prediction (D), the corresponding clinical planned dose ($D_{GT})$, and the prescription dose $\left(\;D_{RP}=7\;\mathrm{or}\;8\;\mathrm{Gy}\;\mathrm{for}\;\mathrm{the}\;\mathrm{test}\;\mathrm{cases}\right)$, and is averaged by the voxel number (n) of the given ROI.

For DVH metrics evaluation, the following metrics were statistically analyzed: $D_{5\%}$ and $D_{95\%}$ for target coverage, and $D_{2\%}$ and $D_{50\%}$ for OARs dose sparing, where $D_{x\%}$ represents the dose that x% of the volume of an ROI is at least receiving. The MAPE for each DVH metric was calculated using D, the DVH metric derived from the smoothed dose distribution prediction, and $D_{GT}$, the DVH metric from the clinically planned dose distribution. The calculation was averaged over the number of test cases (n=64). Also, the Wilcoxon signed‐rank test was used to compare the DVH metrics from smoothed prediction and clinical plans, where the differences were considered statistically significant for p<0.05.

(3) $$\mathrm{MAPE}\;\;\left(\%\right)=\frac{100}{n}\;\sum_{i=1}^{n}\left|\frac{D_{i}-D_{GT_{i}}}{D_{RP}}\right|$$

The GPR is set to 3%/2 mm and 3%/1 mm, respectively, to assess the smoothed prediction with clinically planned dose. The lowest dose predicted by Seg2Dose 5% is 2.5% relative dose, while for Seg2Dose 2%, it is 1% relative dose.

## 3 RESULTS

**FIGURE 3:**
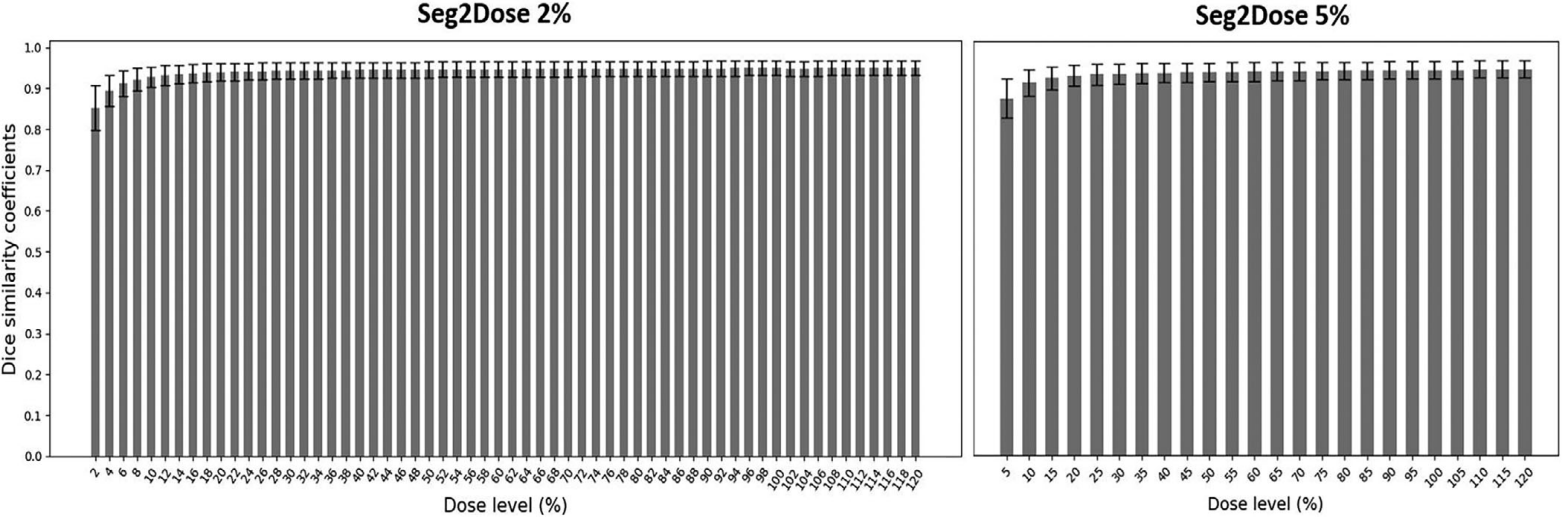
The mean DSCs comparing isodose volumes between the clinical dose and predicted dose from Seg2Dose 2% and Seg2Dose 5% for 64 test cases. The error bar represents 1 standard deviation. DSC, Dice similarity coefficient.

The result of the mean and standard deviation of the DSC for the isodose volumes from 64 test cases of Seg2Dose 2% and Seg2Dose 5% are shown in Figure 3. The DSC value ranges from 0 to 1, with 1 indicating a perfect match. The average mean DSC for the isodose volumes is 0.94 for Seg2Dose 2% and 0.93 for Seg2Dose 5%. The lowest mean DSC for Seg2Dose occurs in the t=1 isodose volume, and all other isodose volumes have a mean DSC above or equal to 0.9. For Seg2Dose 2%, this corresponds to the range [1%, 3%] with a mean DSC of 0.85 ± 0.05, while for Seg2Dose 5%, it corresponds to the range [2.5%, 7.5%] with a mean DSC of 0.87 ± 0.05. With a given 5% interval, relative doses below 2.5% are ignored during the training phase, leading to unstable spatial features in low‐dose regions and making it difficult for Seg2Dose to predict as much details as possible. However, this issue can be mitigated by reducing the interval. In Seg2Dose 2%, the mean DSC for the ranges [1%, 5%] and [1%, 7%] exceeds 0.9.

**FIGURE 4:**
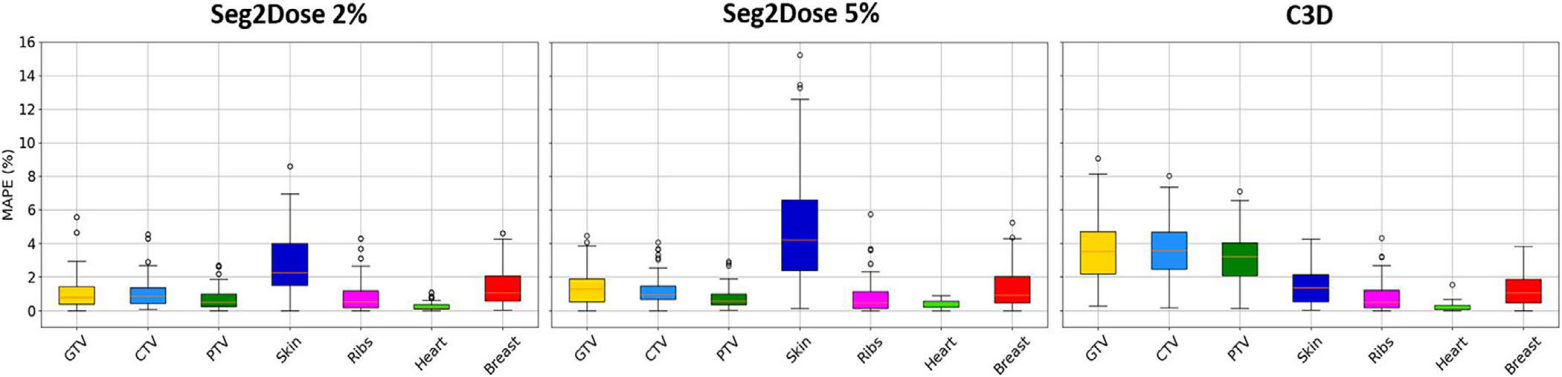
The MAPE statistics for the predictions of Seg2Dose 2%, Seg2Dose 5%, and C3D prediction for the ROI of 64 test patients. C3D, Cascade 3D; MAPE, mean absolute percent error; ROI, region of interest.

Figure 4 presents the box plot of the MAPE of Seg2Dose 2%, Seg2Dose 5%, and C3D for three targets and four OARs for the 64 test cases. Further dosimetric comparisons between predicted dose and clinically planned dose in terms of target coverage and OAR sparing are summarized in Tables 2 and 3. For the Seg2Dose model, the skin contains the highest MAPE at 8.58% for Seg2Dose 2% and 15.25% for Seg2Dose 5%, which also shows significant differences and high MAPE in the dose sparing metrics $D_{2\%}$ and $D_{50\%}$. Other ROIs maintain a high prediction accuracy with low MAPEs within 6%, and their DVH metrics, $D_{5\%}$ and $D_{95\%}$ for targets, and $D_{2\%}$ and $D_{50\%}$ for OARs are also well predicted by the Seg2Dose. For the C3D model, compared to Seg2Dose, the dose prediction in the target area exhibited higher MAPEs and was significantly lower than clinically planned dose. The C3D achieved lower MAPE for skin in both voxel‐based dose and DVH metrics evaluations compared to Seg2Dose.

**TABLE 2:** Target coverage statistical DVH metrics comparison of clinical dose distribution (clinical) and prediction dose distribution (Seg2Dose) among 64 test cases (mean ± std.).

	Prescription dose $D_{95\%}=8\;Gy$ of CTV							
	$D_{5}$				$D_{95}$			
	PTV	MAPE	CTV	MAPE	PTV	MAPE	CTV	MAPE
Clinical	8.93 ± 0.15	–	8.98 ± 0.16	–	7.18 ± 0.08	–	8.01 ± 0.02	–
Seg2Dose 5%	8.87 ± 0.15	1.73%	8.92 ± 0.15	1.93%	7.20 ± 0.02	0.71%	8.00 ± 0.04	0.38%
Seg2Dose 2%	8.88 ± 0.15	1.52%	8.92 ± 0.16	1.55%	7.21 ± 0.07	1.13%	8.00 ± 0.06	0.67%
C3D	**8.51 ± 0.37**	5.64%	**8.53 ± 0.36**	5.84%	**7.05 ± 0.19**	2.29%	**7.79 ± 0.19**	3.18%

	Prescription dose $D_{95\%}=7\;Gy$ of CTV							
	$D_{5}$				$D_{95}$			
	PTV	MAPE	CTV	MAPE	PTV	MAPE	CTV	MAPE
Clinical	7.58 ± 0.15	–	7.61 ± 0.16	–	**6.46 ± 0.11**	–	**7.02 ± 0.02**	–
Seg2Dose 5%	7.63 ± 0.13	2.35%	7.66 ± 0.13	2.35%	**6.38 ± 0.09**	1.74%	**6.99 ± 0.06**	0.62%
Seg2Dose 2%	7.57 ± 0.12	1.73%	7.59 ± 0.13	1.85%	6.44 ± 0.09	1.24%	7.01 ± 0.05	0.60%
C3D	**7.24 ± 0.12**	4.84%	**7.25 ± 0.12**	5.07%	**6.38 ± 0.09**	1.61%	**6.85 ± 0.09**	2.36%

*Note*: The Wilcoxon signed‐rank test is used to compare plans, with significant values marked in bold (p<0.05).

Abbreviations: C3D, Cascade 3D; CTV, clinical target volume; DVH, dose‐volume histogram; MAPE, mean absolute percent error; PTV, planning target volume.

**TABLE 3:** OAR dose statistical DVH metrics comparison of clinical dose distribution (clinical) and prediction dose distribution (Seg2Dose) among 64 test cases (mean ± std.).

	Prescription dose $D_{95\%}=8\;Gy$ of CTV								
		Heart	MAPE	Ribs	MAPE	Skin	MAPE	Breast	MAPE
$D_{2}$	Clinical	0.55 ± 0.28	–	2.27 ± 1.52	–	5.49 ± 0.97	–	8.63 ± 0.11	–
	Seg2Dose 5%	0.54 ± 0.34	1.33%	2.39 ± 1.67	2.10%	**4.64 ± 0.94**	11.02%	8.61 ± 0.14	1.38%
	Seg2Dose 2%	0.55 ± 0.32	0.55%	2.37 ± 1.66	2.08%	**4.83 ± 0.87**	8.40%	8.58 ± 0.11	1.17%
	C3D	0.53 ± 0.28	0.40%	**2.38 ± 1.61**	1.65%	5.35 ± 0.82	3.03%	**8.30 ± 0.21**	4.50%
$D_{50}$	Clinical	0.16 ± 0.09	–	0.17 ± 0.38	–	0.96 ± 0.26	–	1.26 ± 0.40	–
	Seg2Dose 5%	0.18 ± 0.17	1.07%	0.17 ± 0.46	0.59%	**0.89 ± 0.18**	1.38%	1.27 ± 0.37	0.99%
	Seg2Dose 2%	**0.14 ± 0.10**	0.31%	0.20 ± 0.44	0.60%	0.93 ± 0.20	1.32%	1.26 ± 0.37	1.03%
	C3D	**0.14 ± 0.08**	0.21%	**0.19 ± 0.42**	0.27%	1.00 ± 0.16	1.68%	**1.34 ± 0.35**	1.29%

	Prescription dose $D_{95\%}=7\;Gy$ of CTV								
		Heart	MAPE	Ribs	MAPE	Skin	MAPE	Breast	MAPE
$D_{2}$	Clinical	0.76 ± 0.45	–	2.70 ± 1.42	–	5.06 ± 1.04	–	7.41 ± 0.10	–
	Seg2Dose 5%	0.80 ± 0.52	1.42%	2.76 ± 1.38	3.99%	**3.72 ± 0.90**	19.13%	7.44 ± 0.11	1.48%
	Seg2Dose 2%	0.78 ± 0.46	1.12%	2.71 ± 1.39	3.58%	**4.25 ± 0.95**	11.65%	7.41 ± 0.09	1.21%
	C3D	0.77 ± 0.47	1.25%	2.77 ± 1.35	2.97%	**4.98 ± 0.87**	3.55%	**7.14 ±** **0.11**	3.80%
$D_{50}$	Clinical	0.12 ± 0.06	–	0.81 ± 0.36	–	1.05 ± 0.30	–	1.52 ± 0.49	–
	Seg2Dose 5%	0.10 ± 0.14	1.23%	0.81 ± 0.38	0.90%	**0.85 ± 0.15**	3.13%	**1.45 ± 0.39**	2.07%
	Seg2Dose 2%	0.12 ± 0.08	0.54%	0.81 ± 0.34	0.68%	**0.96 ± 0.24**	1.82%	1.47 ± 0.40	1.68%
	C3D	**0.11 ± 0.04**	0.24%	**0.85 ± 0.39**	0.87%	**1.10 ± 0.28**	1.30%	**1.61 ± 0.46**	1.71%

*Note*: The Wilcoxon signed‐rank test is used to compare plans, with significant values marked in bold (p<0.05).

Abbreviations: C3D, Cascade 3D; CTV, clinical target volume; DVH, dose‐volume histogram; MAPE, mean absolute percent error; OAR, organ at risk.

**FIGURE 5:**
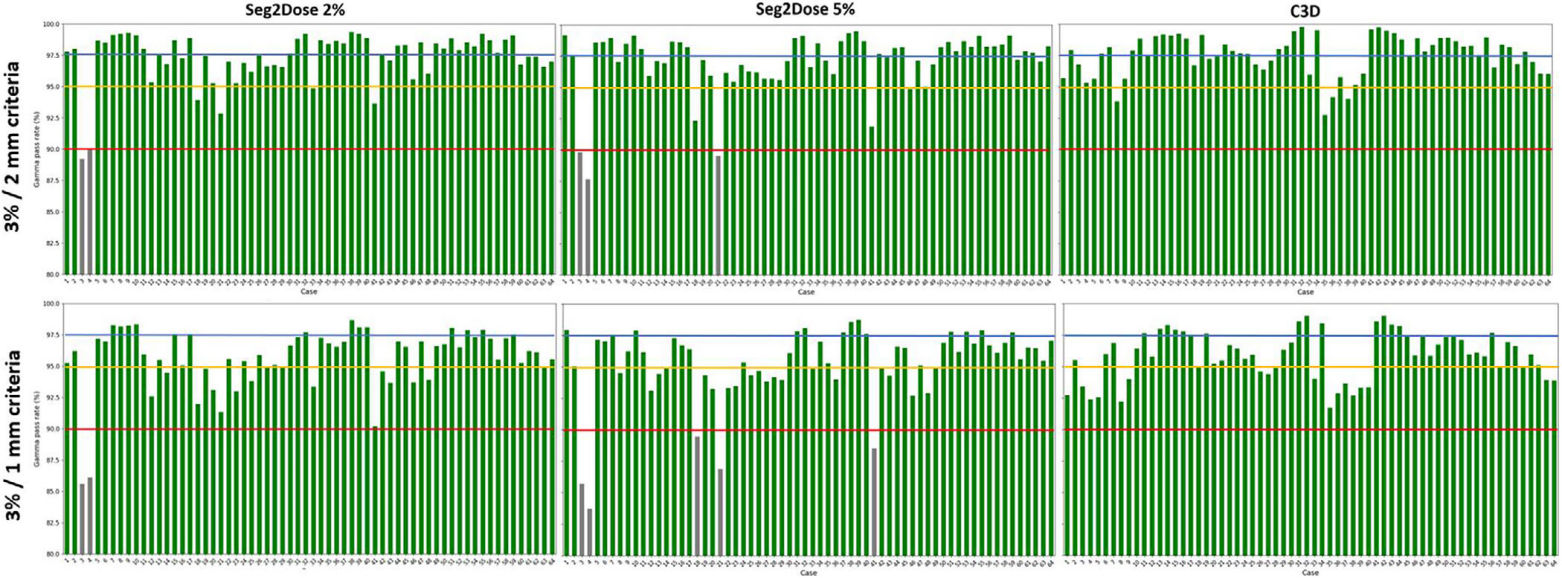
The global GPRs (3%/2 mm and 3%/1 mm criteria GPR) of 64 test cases predicted by Seg2Dose 2%, Seg2Dose 5%, and C3D, with 90%, 95%, and 97.5% GPR are represented by red, yellow, and blue lines, respectively, and cases with GPR below 90% are colored in gray. C3D, Cascade 3D; GPR, gamma passing rate.

Figure 5 illustrates the 3%/2 mm and 3%/1 mm GPR results for each test case by Seg2Dose 2%, Seg2Dose 5%, and C3D. The percentages of cases passing the 90%, 95%, and 97.5% thresholds for 3%/1 mm GPR were summarized in Table 4. The C3D model has a better performance in GPR evaluation than Seg2Dose models, with 100% cases passing 90% thresholds of both 3%/2 mm and 3%/1 mm GPR. As the criteria become stricter, only Seg2Dose 5% shows an additional two cases falling below the 90% threshold for GPR. The below‐threshold cases are attributed to the presence of hotspots in the target area in the clinical plans or variations in the prescription dose across the 5‐fraction treatment, such as the prescription dose for the first fraction was set to 7 Gy while the remaining fractions were adjusted to 8 Gy to provide better control of the tumor. The lowest observed global GPR for Seg2Dose 5% was 87.66% under the 3%/2 mm criteria and 83.73% under the 3%/1 mm criteria.

**TABLE 4:** The percentages of cases passing the 90%, 95%, and 97.5% thresholds for 3%/1 mm GPR of Seg2Dose 2%, Seg2Dose 5%, and C3D.

Passing X% GPR	Seg2Dose 2%	Seg2Dose 5%	C3D
90% GPR	96.9% (62/64)	92.1% (59/64)	100% (64/64)
95% GPR	75.0% (48/64)	65.6% (42/64)	71.9% (46/64)
97.5% GPR	21.9% (14/64)	20.3% (13/64)	26.6% (17/64)

Abbreviations: C3D, Cascade 3D; GPR, gamma passing rate.

**FIGURE 6:**
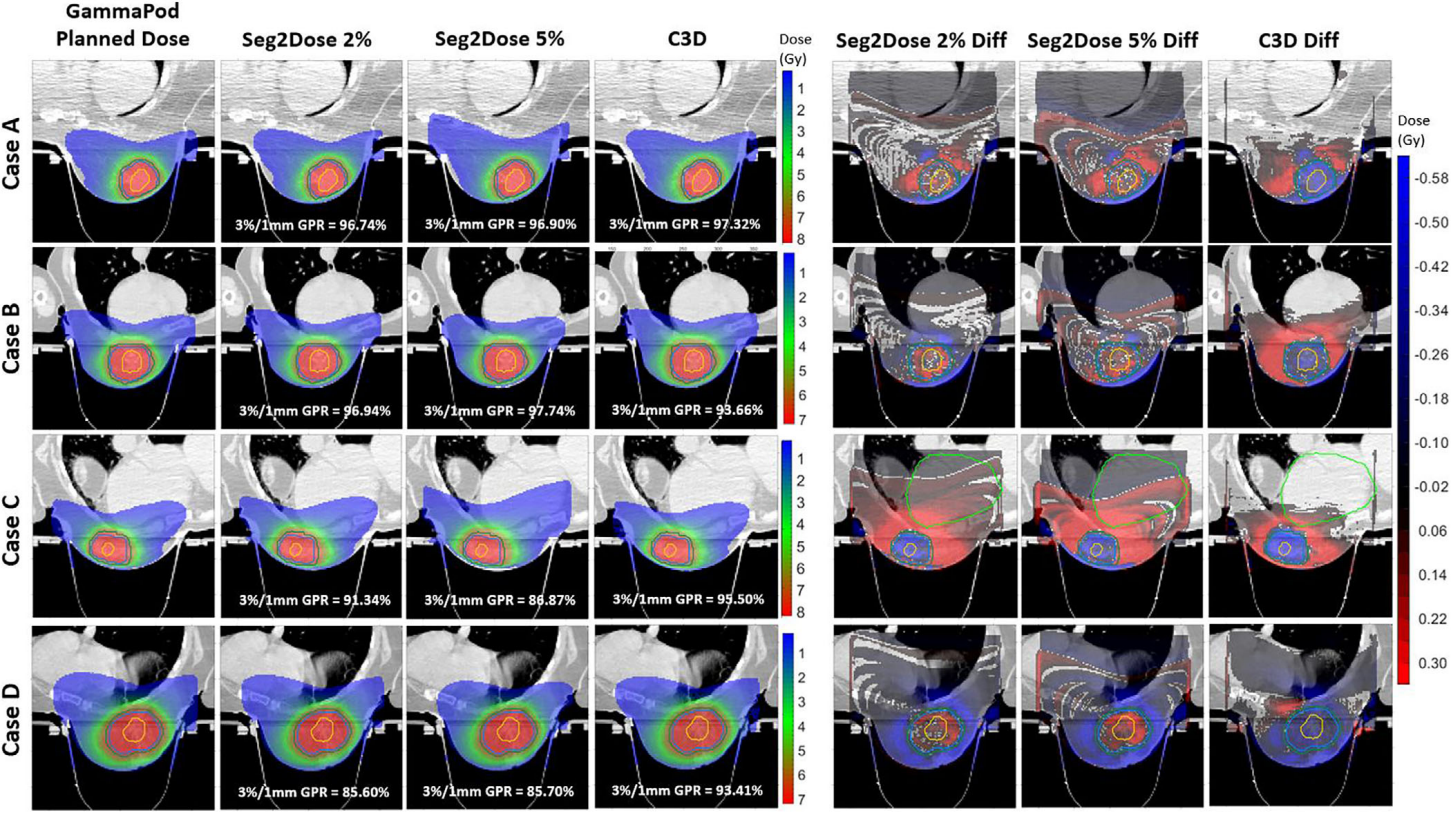
Comparison of dose distribution and dose difference (smoothed prediction—GammaPod planned dose) between clinical plans (GammaPod), Seg2Dose 2%, Seg2Dose 5%, and C3D predictions for four cases. Cases A and B represent the normal case with over 90% GPR, and cases C and D represent the worst case with GPR below 90%. The yellow, blue, and green segmented structures are GTV, CTV, and PTV, and the light green segmented structure in dose difference is the heart. C3D, Cascade 3D; CTV, clinical target volume; GPR, gamma passing rate; PTV, planning target volume.

For the case study, we selected two cases, A and B, as normal cases with GPR values above the 90% threshold and different prescription doses: 8 Gy for case A and 7 Gy for case B. cases C and D represent the worst case scenarios, with GPR values below the 90% threshold, also featuring different prescription doses among the 5‐fraction treatments for the same patient. Specifically, for case C, the analyzed fraction has a prescription dose of 8 Gy, while the other four fractions for the same patient were prescribed 7 Gy. Conversely, for case D, the analyzed fraction has a prescription dose of 7 Gy, with one of the other fractions prescribed 8 Gy. The dose distribution and dose difference with clinical planned dose for cases A–D by Seg2Dose and C3D models were shown in Figure 6. The predictions from Seg2Dose 2% and C3D are more similar to the clinically planned dose, showing smaller dose differences compared to the Seg2Dose 5% prediction, particularly in the low‐dose region. The areas with large dose gradients are prone to have relatively large errors in both Seg2Dose and C3D models; however, Seg2Dose 2% effectively mitigates this issue by retaining more detailed dose information compared to Seg2Dose 5%. In the worst case scenarios, the relatively higher dose differences observed in Seg2Dose predictions are attributed to the variation in prescription doses across fractions for the same patient. Specifically, for case C, the clinical plan reflects a more rapid dose fall‐off to achieve similar OAR sparing due to the increased prescription dose for that fraction. This results in a lower dose outside the target in the clinical dose distribution, creating a mismatch with Seg2Dose predictions. For case D, the opposite scenario is observed. Additionally, we have included a case study in Figure S1 to demonstrate the performance of Seg2Dose for a patient with a consistent prescription dose across a 5‐fraction treatment, using Seg2Dose 5% as an example. Moreover, even when the 3%/1 mm GPR is close to or above 90%, the voxel‐wise dose difference of case C reveals high dose predictions near and within the breast in both the Seg2Dose and C3D models. Additionally, most voxel‐wise dose predictions in the target area by the C3D model are lower than the clinical dose in both normal and worst cases.

## 4 DISCUSSION

In this study, we propose a novel and generic strategy, Seg2Dose, which utilizes the segmentation network as an off‐the‐shelf 3D dose prediction tool without revising the model architecture. The performance and feasibility of Seg2Dose with different training and inference dose level intervals (2% and 5%) were evaluated on a cohort of breast cancer patients treated with GammaPod and compared with a cutting‐edge continuous dose prediction model, C3D. The results demonstrate that both the dose levels and processed smoothed dose distributions predicted by Seg2Dose achieved high accuracy compared to clinical plans. Furthermore, with finer dose level intervals, Seg2Dose achieved comparable prediction accuracy to the C3D model while offering distinct advantages for integration into clinical workflows. These findings suggest that Seg2Dose has the potential to be developed into a plug‐in application, providing quick dose or DVH previews for patients as soon as images are scanned and ROIs are contoured.

To improve the performance of dose prediction models, integrating advanced network architectures, such as generative adversarial networks (GANs) or diffusion models, to extract more features has been shown to be effective.^24^, ^25^, ^26^, ^27^ However, these approaches often come with significant challenges, including training instability for GANs, the computationally expensive iterative inference process for diffusion models, and the need for careful tuning of parameters in both models. Additionally, these generative networks require expert knowledge for architectural design and task‐specific modifications, which increases the implementation complexity and limits their adaptability to diverse clinical scenarios. In contrast, Seg2Dose is specifically designed to balance implementation simplicity and prediction accuracy. The Seg2Dose framework does not aim to provide precise guidance for dose optimization or calculation but instead offers a quick and efficient overview of the dose distribution for a specific treatment modality. This overview helps physicists gain an initial understanding of the expected dose distribution before conducting the detailed planning process.

Recent studies have found that proper data preparing, preprocessing and training techniques are more important to unlock the potential of CNN networks than architectural modifications.^15^, ^21^ Leveraging the Adapter module, Seg2Dose processes clinical datasets into a format suitable for segmentation networks with an encoder–decoder architecture. The Smoother module then converts the discrete dose predictions into continuous distributions. This modular design allows Seg2Dose to operate as an out‐of‐the‐box solution without requiring architectural modifications or extensive parameter tuning, making it significantly easier to implement in clinical workflows. By streamlining the dose prediction process and focusing on usability, Seg2Dose represents a practical alternative to generative models, offering a faster, simpler, and more accessible tool for radiotherapy dose prediction while maintaining reasonable prediction accuracy.

For the Seg2Dose strategy, a major concern may arise regarding the potential reduction in prediction accuracy when using the dose level map as the ground truth, which simplifies the continuous dose distribution into a discrete pattern. To address this, the C3D model, trained on the continuous dose distribution, was adopted as a baseline to evaluate the impact on prediction accuracy. Based on the statistical results, including MAPE and DVH metrics, as well as the case study presented in Figure 6, both the Seg2Dose 2% and Seg2Dose 5% models achieve comparable performance to the C3D model. We hypothesize that the reason behind this is that the dose level map effectively captures the general dose patterns characteristic of the same type of cases treated with the same modality, while voxel‐based dose data are affected by inconsistencies from manual planning and perturbations caused by hot and cold spots. One limitation of Seg2Dose is the Seg2Dose 5% model shows lower prediction accuracy in the low‐dose region and the skin area. This limitation is inherent to dose‐level‐based models, as the condensed dose level map ignores intra‐level dose heterogeneity and excludes doses below the lowest dose level threshold. For instance, during the training phase, the Seg2Dose 5% model disregards relative doses below 2.5%, while the Seg2Dose 2% model omits relative doses below 1%. Completely eliminating this limitation, that is, enabling Seg2Dose to train on continuous data, would require significant modifications to the loss function and model architecture of nnU‐Net, which is beyond the scope of this study. The current results indicate that reducing the interval between dose levels offers a potential alternative to addressing this issue, as it improves prediction accuracy not only in the skin and low‐dose regions but also in other ROIs, albeit at the cost of longer training time to achieve a converged loss score. Another limitation of Seg2Dose, demonstrated in the worst case scenarios shown in Figure 6, is that relying solely on the prescription dose and avoidance mask restricts the ability of Seg2Dose to accurately infer dose distributions for the same patient with different prescriptions.

Apart from differences in the ground truth patterns, the implementation of Seg2Dose and C3D differs in two key aspects. The first is the automated configuration characteristic of nnU‐Net,^21^ which starts with the extraction of the dataset fingerprint and subsequent execution of heuristic rules. This automated process covers the entire segmentation pipeline without requiring any user intervention, allowing the model to adapt to new datasets with robust performance. In contrast, the preprocessing and data augmentation steps in the C3D model are predefined and cannot automatically adapt to new datasets. The second is the design of input channel. Unlike C3D model that process OAR independently and use CT image to assist dose prediction, Seg2Dose only takes the prescribed dose and avoidance mask which encompasses the OARs with different weights as two input channels. This is due to the fact that the anatomical information from OARs and the prescribed dose are the most important factors affecting dose distribution.^28^ This channel‐fixed design enables Seg2Dose to function as an out‐of‐the‐box tool, whereas the C3D model requires manual adjustments. Also, both C3D model and an experiment exploring the impact of input data on dose prediction indicated that CT scans give limited additional benefit when OARs were used.^15^, ^19^ Therefore, we excluded CT from the input data, but further study can be conducted by adding CT as an input channel to investigate the impact of different inputs. The weight assignment for each OAR in the avoidance mask follows the general clinical object priorities for treatment planning, enabling the model to account for the varying levels of protection required by different regions and ensuring that the predicted dose aligns with clinical planning objectives. In contrast, conventional models like C3D typically treat each OAR as an individual binary mask, lacking the capability to consider the differing sensitivities among OARs. We also investigated the impact of using equal weight assignment during the inference stage for Seg2Dose 5% as an example, which resulted in an increased prediction MAPE for most ROIs, as shown in the Figure S2.

Moreover, the current Smoother module primarily smooths the discrete values using the Gaussian kernel with a fixed bandwidth, which is a common and effective method but is prone to yield errors in the isodose volume where the dose falls rapidly. For example, the poor predictive performance of skin indicated by MAPE and dose metrics $D_{2\%}$ and $D_{50\%}$ is due to the fact that skin is located in a region close to the targets and the body surface, where dose distribution typically changes rapidly. Although the inherent issue discussed above can be mitigated by reducing the dose level interval, we also demonstrate the post‐processing effect by quantifying the impact of the Gaussian filter on prediction accuracy and comparing it with an interpolation‐based smoothing method, as presented in Figures S3–S5. Specifically, the MAPEs for each ROI and the 3%/2 mm GPR with clinical plans were comparable among Seg2Dose 5%, the dose distribution with the Gaussian filter, and the dose distribution with the interpolation‐based method, with minor variations across different ROIs. As an alternative smoothing approach, a neural network could be trained to learn the mapping from dose levels to dose distributions.^29^ Additionally, it is worth noting that the 3D nnU‐Net is not the only possible foundation for Seg2Dose; other 3D segmentation networks could be considered as substitutes.

While the current study only confirms the feasibility of Seg2Dose in GammaPod therapy, this dose prediction strategy is promising for broader applications in volumetric modulated arc therapy (VMAT), brachytherapy, and proton therapy. Using finer and suitable dose level intervals may improve prediction accuracy in regions with sharp dose gradients. However, further validation and evaluation are essential to establish its efficacy and reliability in these additional treatment modalities, such as the ability to incorporate beam configuration features for variable beam setups. Further, Seg2Dose can assist in the treatment modality selection process. The challenge of applying Seg2Dose to multiple treatment modalities lies in the collection of sufficient high‐quality datasets, mainly a matter of time. To further explore the potential of Seg2Dose as a plug‐in quick dose previewer, planners can adjust target contour delineations, weight assignments for OARs in the avoidance area, and/or prescription dose preferences to compare the resulting dose changes. This approach can provide planning guidance for new patients, potentially improve plan quality, reduce planning time, and eliminate planning variability between centers and physicians.

## 5 CONCLUSION

In this study, we introduced Seg2Dose, a novel strategy that leverages segmentation networks for 3D dose prediction without the need for customizing model architecture. Evaluated on a dataset of breast cancer patients treated with GammaPod, Seg2Dose demonstrated high accuracy in dose level maps and processed dose distributions compared to clinical plans. Its primary advantage lies in functioning as an off‐the‐shelf tool, simplifying input requirements by considering only the prescribed dose and avoidance regions. By providing a rapid dose preview tool, Seg2Dose has the potential to improve planning quality, reduce planning time, and hopefully support optimal radiotherapy technology selection in the future.

## CONFLICT OF INTEREST STATEMENT

The authors declare no conflicts of interest.

## Supporting information



Supporting Information
