# Supplementary material for: Radiotherapy dose prediction using off‐the‐shelf segmentation networks: A feasibility study with GammaPod planning
Source: Med Phys. 2025 Feb 28;52(5):3348–59. doi: 10.1002/mp.17711 (PMC12059546; doi:10.1002/mp.17711)
Supplement: Supplementary file 1 — Supporting Information [file MP-52-3348-s001.docx]

# Supplementary Material

This document contains supplementary results to accompany the manuscript named: “Radiotherapy Dose Prediction Using Off-the-Shelf Segmentation Networks: A Feasibility Study with GammaPod Planning”.

## Case study: same patient with a 5-fraction treatment

An example of dose distributions predicted by Seg2Dose 5% for a same patient with 5-fraction treatment is displayed in Figure S-1. The top row shows two input channels superimposed in a single diagram. The clinical dose distributions are shown in the second row, and the dose difference is calculated by the Seg2Dose 5% dose distribution minus the clinical dose distribution. Among the 5-fraction plans, the absolute dose differences remained within 1 Gy, and the 3%/2 mm GPR exceeded 96% for all cases. The areas with large dose gradients are prone to have relatively large errors, as seen in the dose difference results shown in the first and second fractions.

|  |
| --- |

1. Ablation study: Different weight assignments in Avoidance channel

As mentioned in the discussion, we performed an ablation study to investigate the impact of different weight assignments on the Seg2Dose 5% prediction results. Table S-1 shown two different weight assignments. Weights A represents the weight assignments adopted in the Seg2Dose 5% model, Weight B was only used for Seg2Dose 5% testing stage, which assumes equal importance for all ROIs. The predicted dose distributions using Weight B showed an increased prediction MAPE for most ROIs, except for the skin.

## The impact of the Gaussian filter and interpolation-based smoothing method

Using the Seg2Dose 5% as an example model, Figure S-3 demonstrates the post-processing effect on the nnU-Net dose prediction. The dose distribution in Figure S-3 (b) is generated by converting the dose-level map with discrete isodose indicators (Figure S-3 (a)) into absolute isodose volumes and smoothing them with a Gaussian filter, which has a bandwidth of 2 mm to control the degree of smoothing. The dose distribution in Figure S-3 (c) is generated by using linear interpolation method to simulate the continuous dose drop-off between the isodose indicators in the Figure S-3 (a). Figure S-3 (d) compared the smoothing effect on the DVH between dose level and the dose distributions smoothed by Gaussian filter and interpolation, respectively. Figure S-4 and Figure S-5 compare the impact of Gaussian filter and interpolation method on the Seg2Dose 5% prediction accuracy, evaluating by the MAPE and GPR. Overall, the smoothing effects of both methods are comparable, although their impact varies across different ROIs.

$$MAPE \left( \% \right)=\frac{100}{n}\sum_{i=1}^{n} \left| \frac{D_{i}-D_{{GT}_{i}}}{D_{RP}} \right|$$

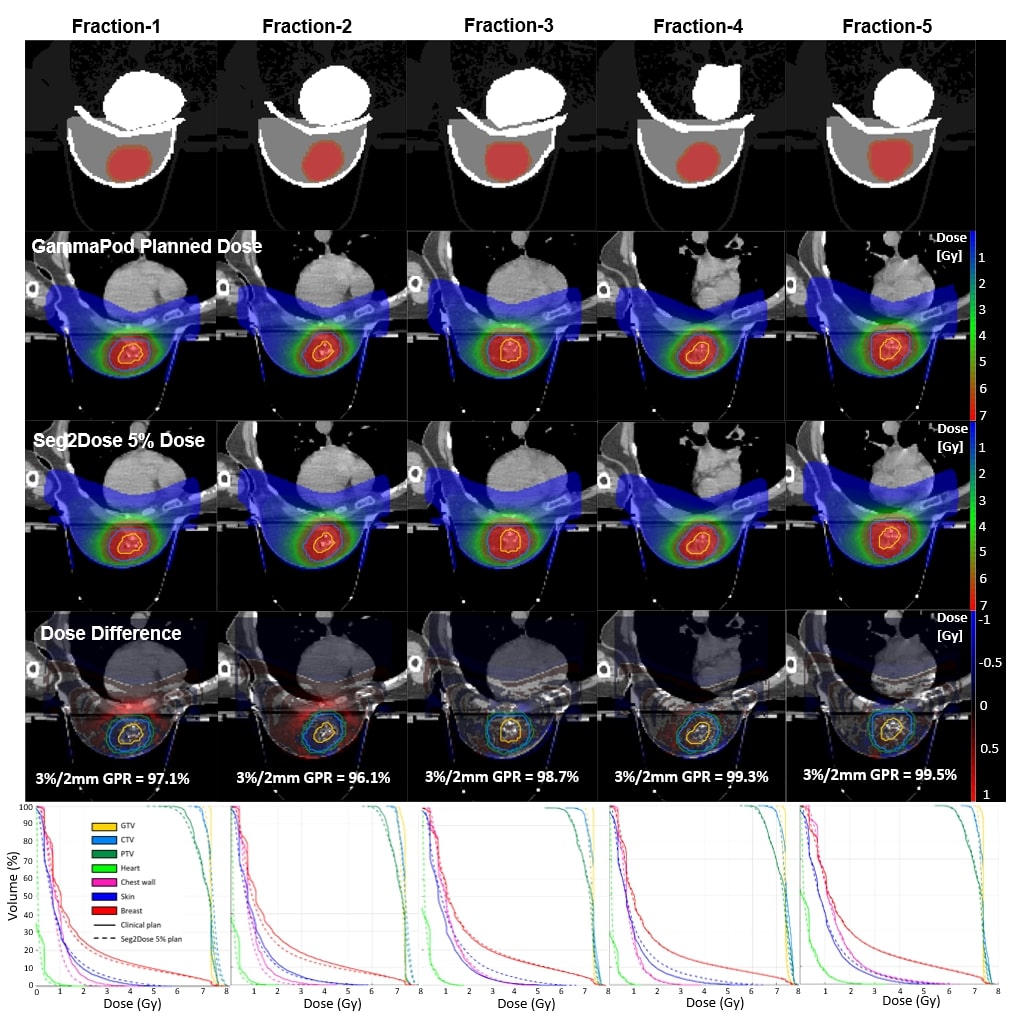


Figure S-1. Comparison of dose distribution overlaid on the CT scan and DVH between clinical plans (GammaPod) and Seg2Dose 5% predictions for a same patient with 5-fractions. The input channels superpose avoidance and prescription dose together. The yellow, blue and green segmented structure are GTV, CTV and PTV. The dose difference (Seg2Dose - GammaPod Planned Dose) is shown with corresponding 3%/2mm gamma passing rate.


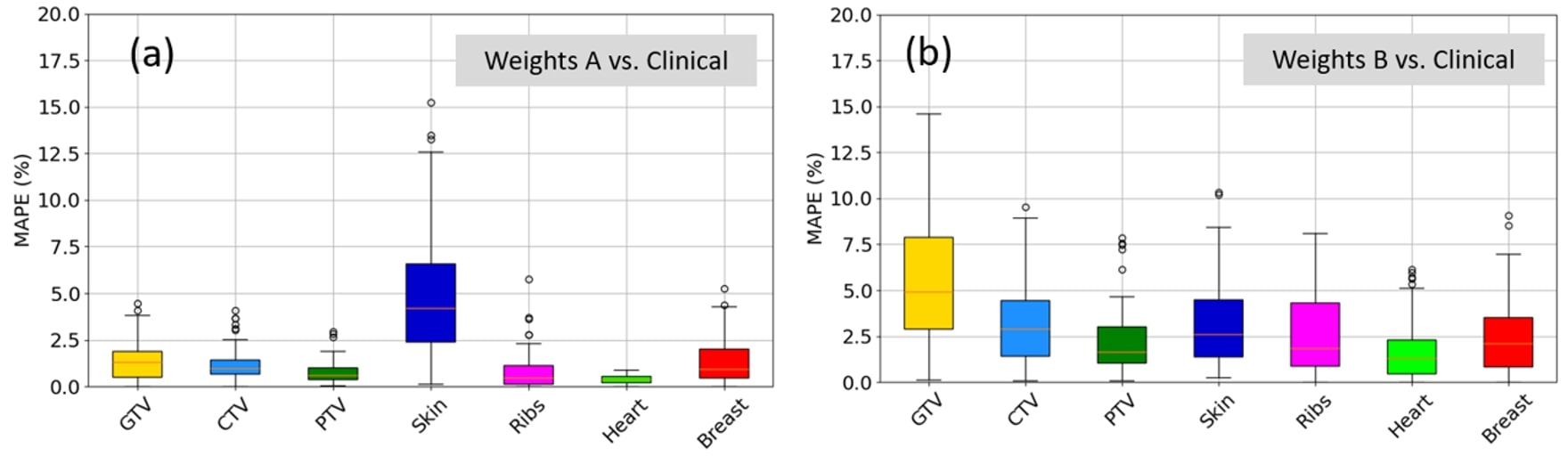


Figure S-2. The mean absolute percent error (MAPE) statistics between clinical planned dose and smoothed Seg2Dose 5% prediction for the regions of interest (ROIs) of 64 test patients. (a) represents the MAPE of the clinical planned dose and smoothed Seg2Dose 5% dose distribution prediction with Weights A assignment (same sub-figure in Figure 4 Seg2Dose 5%). Similar MAPE results for (b) with different weight assignments Weights B.


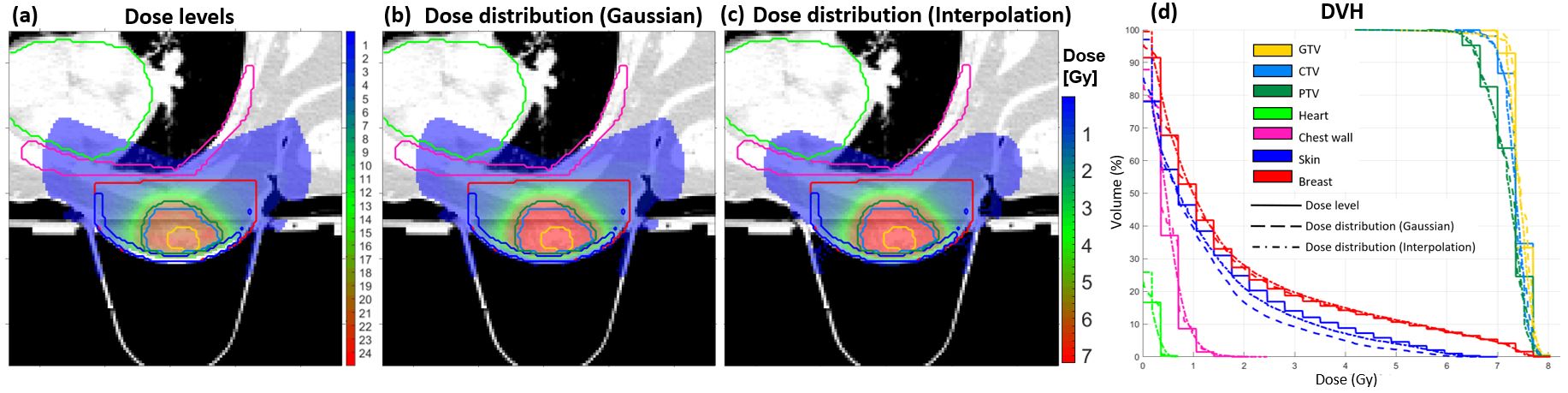


Figure S-3. The post-processing effect on prediction of Seg2Dose 5%, (a) the dose level prediction of Seg2Dose 5%, (b) the dose distribution after post-processing by Gaussian kernel, (c) the dose distribution after post-processing by linear interpolation, (d) the DVH comparison of dose level distribution and dose distributions processed by Gaussian kernel and linear interpolation of Seg2Dose 5%.


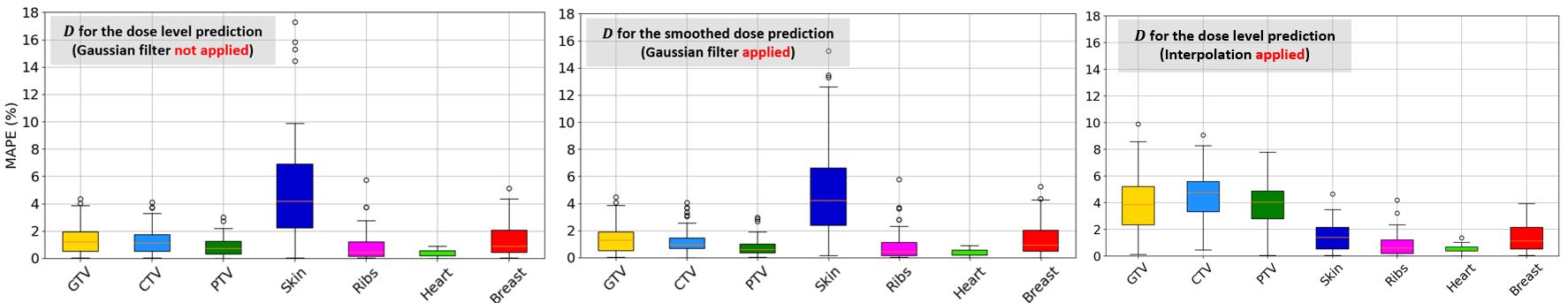


Figure S-4. The mean absolute percent error (MAPE) statistics for the predictions of Seg2Dose 5% not applied smoothing process and applied the Gaussian filter or linear interpolation for the regions of interest (ROIs) of 64 test patients.


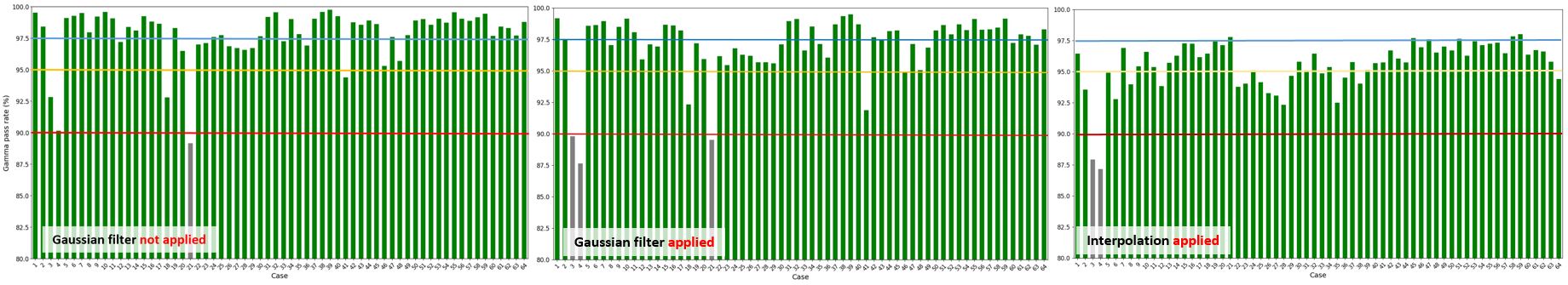


Figure S-5. The global gamma passing rates (3%/2 mm GPR criteria) for 64 test cases predicted by Seg2Dose 5%, without smoothing process and with the application of the Gaussian filter or linear interpolation, are shown. The 90%, 95%, and 97.5% GPR thresholds are represented by red, yellow, and blue lines, respectively, while cases with GPR below 90% are highlighted in gray.

Table S-1. Two different weight assignments in Avoidance channel. Weights A is the weight assignment adopted by current Seg2Dose 5% model during both training and testing stages, while Weights B was applied solely for Seg2Dose 5% testing.

| ROI name | Weights A | Weights B |
| --- | --- | --- |
| Heart | 1 | 1 |
| Skin | 1 | 1 |
| Ribs | 1 | 1 |
| Breast | 0.5 | 1 |
| Body | 0.1 | 1 |
